# Commentary: An ounce of prevention may not be worth a pound of cure—in postoperative atrial fibrillation

**DOI:** 10.1016/j.xjon.2020.08.005

**Published:** 2020-08-08

**Authors:** Magdy M. El-Sayed Ahmed, Kevin P. Landolfo

**Affiliations:** a Department of Cardiothoracic Surgery, Mayo Clinic, Jacksonville, Fla; b Department of Surgery, Zagazig University Faculty of Human Medicine, Zagazig, Egypt

**Figure:**
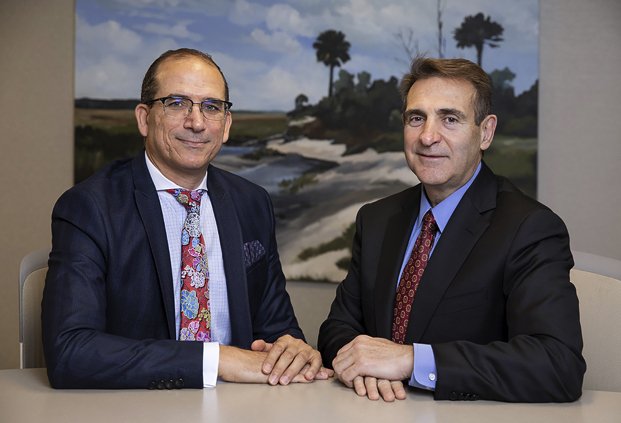
Magdy M. El-Sayed Ahmed, MD, MS (*left*), and Kevin P. Landolfo, MD, MS (*right*)

**Central Message**

Beta-blocker administration may reduce the incidence of isolated postoperative atrial fibrillation.

See Article page 66.

Atrial fibrillation (AF) is the most common atrial arrhythmia, affecting 33 million worldwide.^1^ Moreover, it is the leading cause of hospitalization for arrhythmias.^2^, ^3^, ^4^ Post-cardiac surgery, the incidence of AF (postoperative atrial fibrillation [POAF]) is reported to be 25% to 40%, posing increased risk for stroke, sepsis, prolonged hospital length of stay, greater hospital costs, as well as being a marker for increased mortality.^5^^,^^6^

Numerous pharmacologic agents have been purported to decrease the incidence of POAF. Despite a paucity of conclusive evidence, preoperative administration of β-blockers within 24 hours before revascularization (coronary artery bypass grafting [CABG]) is a Class I recommendation and remains a CABG quality indicator in the US national Society of Thoracic Surgeons registry.^7^^,^^8^

In the current issue of the *Journal*, Masuda and colleagues^9^ report a meta-analysis of randomized controlled trials (RCTs) on the effect of perioperative β-blocker administration on isolated POAF following cardiac surgery. Their study was conducted in accordance with the Preferred Reporting Items for Systematic Reviews and Meta-Analyses guidelines. The authors identified 17 RCTs (1983-2020) and included a total of 650 patients treated with β-blocker and 711 control patients. The majority of patients underwent CABG (87% treatment, 86% control). The most important finding of the analysis was a 48% reduction of risk of “isolated” POAF in the β-blocker–treated patients following cardiac surgery—although this notable difference was not statistically significant. The current analysis is consistent with previous studies, including a metanalysis of RCT published by Khan and colleagues^10^ demonstrating a 51% reduction in POAF following cardiac surgery.

Subgroup analysis from the study demonstrated differences in POAF between type of operation performed, timing of drug administration, specific β-blocker used, and route of administration, although no statistically significant differences were observed. The analysis included patients treated with the ultrashort acting β-blocker landiolol hydrochloride (2 μg/kg/min intravenous administration), the most effective drug in reducing POAF in the study. Landiolol hydrochloride was introduced in Japan and increasingly is prescribed in Europe in the operative and intensive care unit settings for the treatment and prevention of AF.^11^ Limited experience in post-cardiac surgery patients precludes recommendation for widespread adoption of landiolol hydrochloride as a preferred medical treatment for POAF, but further study is warranted.

Overall, the analysis confirms the effectiveness of β-blockers in reducing isolated POAF but fails to clarify best clinical practices for drug administration. The current study supports current national guidelines without adding further clarification for practicing clinicians.

“An ounce of prevention may be worth a pound of cure”—however, the evidence for prophylactic β-blocker administration to prevent POAF remains inconclusive. Further study is required to determine the overall effectiveness, timing, and recommended drug of choice. Perioperative β-blocker administration in the setting of cardiac surgery remains a recommended “best practice,” although the specifics of treatment remain elusive.
